# A New Species of Tiger Pleco *Panaqolus* (Siluriformes: Loricariidae) from the Xingu Basin, Brazil

**DOI:** 10.1371/journal.pone.0165388

**Published:** 2016-11-09

**Authors:** Christian Andreas Cramer, Leandro Melo de Sousa

**Affiliations:** 1 Center for Human Genetics, Faculty of Medicine, Philipps-University Marburg, Marburg, Germany; 2 Laboratório de Ictiologia e Pesca, Departamento de Biologia, Universidade Federal de Rondônia, Porto Velho, Rondônia, Brazil; 3 Laboratório de Ictiologia, Faculdade de Ciências Biológicas, Universidade Federal do Pará, Altamira, Pará, Brazil; Laboratoire de Biologie du Développement de Villefranche-sur-Mer, FRANCE

## Abstract

*Panaqolus tankei* is described from the Xingu River, Brazil. The new species is diagnosed from *P*. *albomaculatus*, *P*. *dentex*, *P*. *nix*, *P*. *nocturnus*, and *P*. *koko* by its color pattern consisting of dark and light diagonal bars on the body and bands on the fins (vs. body and fins without bars or bands); from *P*. *albivermis*, *P*. *maccus*, and *P*. *purusiensis* by the width of the dark bars being more or less the same of the light bars (vs. dark bars at least two or three times wider than light bars) and from *P*. *changae* by the absence of vermiculation on the head (vs. vermiculation present on head). The new species differs from *P*. *gnomus* by the orientation of the bars from posterodorsal to anteroventral direction (vs. anterodorsal to posteroventral direction), and from *P*. *claustellifer* by the orientation of the bands in the dorsal fin that are not parallel to the margin (vs. parallel to the margin). The barcoding region (COI) was sequenced for the new species, sequences were deposited in GenBank and were compared with congeners from other drainages. With regard to the current construction of a hydroelectric power plant (a so-called mega dam) in the Xingu River, herewith we increase knowledge of the river Xingu’s ichthyofauna and, thus improve the assessment of the impacts of that construction on the river.

## Introduction

After some debate [1,2,3,4,5,6], *Panaqolus* Isbrücker & Schraml (2001) is now a well-accepted genus easily separated from *Panaque*, the former genus of the *Panaqolus*-species described before 2001, by the absence of a ventrolateral keel on the caudal peduncle (vs. present) and postero-dorsal margin of the supraoccipital plate pointed (vs. margin straight). Currently, eleven species are recognized: *Panaqolus albivermis* Lujan, Steele & Velasquez, 2013; *P*. *albomaculatus* (Kanazawa, 1958), *P*. *changae* (Chockley & Armbruster, 2002), *P*. *claustellifer* (Tan, Souza & Armbruster, 2016), *P*. *dentex* (Günther, 1868), *P*. *gnomus* (Schaefer & Stewart, 1993), *P*. *koko* Fisch-Muller & Covain, 2012, *P*. *maccus* (Schaefer & Stewart, 1993), *P*. *nix* Cramer & Rapp Py-Daniel, 2015, *P*. *nocturnus* (Schaefer & Stewart, 1993), and *P*. *purusiensis* (Lamonte, 1935). The diversity of the genus is, however, nowhere near fully described. There are at least 10 undescribed species known in the aquarium trade from the main drainages of the Amazon, including Río Ucayali, Rio Negro, Rio Madeira, Rio Tapajós, Rio Curuá-Una, Rio Paru and Rio Xingu, amongst others. These species are still awaiting formal description, mainly due to a lack of specimens deposited in scientific collections.

The Rio Xingu, the fourth largest drainage on the Amazon, has an astonishing diversity of loricariids with at least 60 species from more than 25 genera occurring in its Lower and Middle courses, of which 18 species are endemic and 15 are still undescribed (LMS, pers. obs.; [7]).

At the time of writing, a huge hydroelectric power plant is under construction in the middle reaches of the Rio Xingu, with yet unknown consequences for the often highly specialized local fish fauna. Thus, the aim of the present study is to contribute to the effort of improving our knowledge of these endemic fishes by describing a new species of *Panaqolus* from the Lower Xingu River.

## Methods

### Morphological analysis

Counts, measurements and terminology follow Lujan *et al*. [8]. All measurements were made to the nearest 0.1 mm with the use of digital calipers. The following abbreviations were used: SL, standard length; HL, head length; and c&s, cleared and stained. For clearing and double staining we followed the protocol from Tayler & van Dyke [9]. Institutional abbreviations are as listed in Sabaj Pérez [10]. Specimens were collected with LMS’ IBAMA permit #31089–1 which is valid in the whole Brazilian territory. Collected fish were first euthanized using clove oil and then fixed in formalin, following the guidelines of the Brazilian Society of Ichthyology [11]. No endangered species were collected. Only fixed museum material was used for the present work, therefore, according to the Brazilian legislation, no approval by an ethics committee was necessary.

The type specimens were publicly deposited in the permanent collections of the following institutions: INPA, Instituto Nacional de Pesquisas da Amazônia, Manaus/AM, Brazil; USNM, Smithsonian Institution National Museum of Natural History, Washington, D.C., United States of America (USA); MNRJ, Museu Nacional da Universidade Federal do Rio de Janeiro, Rio de Janeiro/RJ, Brazil; MPEG, Museu Paraense Emílio Goeldi, Belém/PA, Brazil; ANSP, Academy of Natural Sciences of Drexel University, Philadelphia, USA; MZUSP, Museu de Zoologia da Universidade de São Paulo, São Paulo/SP, Brazil.

### DNA sequencing, editing, and alignment

Total DNA was extracted from ethanol-preserved fin samples using DNeasy Tissue Kit (Qiagen), following manufacturer’s instructions. Partial sequences of Cytochrome c Oxidase subunit 1 [12] were amplified and sequenced using the protocol and the primers from Roxo *et al*. [13]. The DNA sequences were edited and aligned using BioEdit 7.0.9 [14]. Nucleotide variation was examined using MEGA 6.0 [15].

### Nomenclatural Acts

The electronic edition of this article conforms to the requirements of the amended International Code of Zoological Nomenclature, and hence the new names contained herein are available under that Code from the electronic edition of this article. This published work and the nomenclatural acts it contains have been registered in ZooBank, the online registration system for the ICZN. The ZooBank LSIDs (Life Science Identifiers) can be resolved and the associated information viewed through any standard web browser by appending the LSID to the prefix “http://zoobank.org/”. The LSID for this publication is: urn:lsid:zoobank.org:pub:F416B09C-018D-441F-88F5-9284A1330EF9. The electronic edition of this work was published in a journal with an ISSN and has been archived and is available from the following digital repositories: PubMed Central, LOCKSS.

## Results

**Fig 1:**
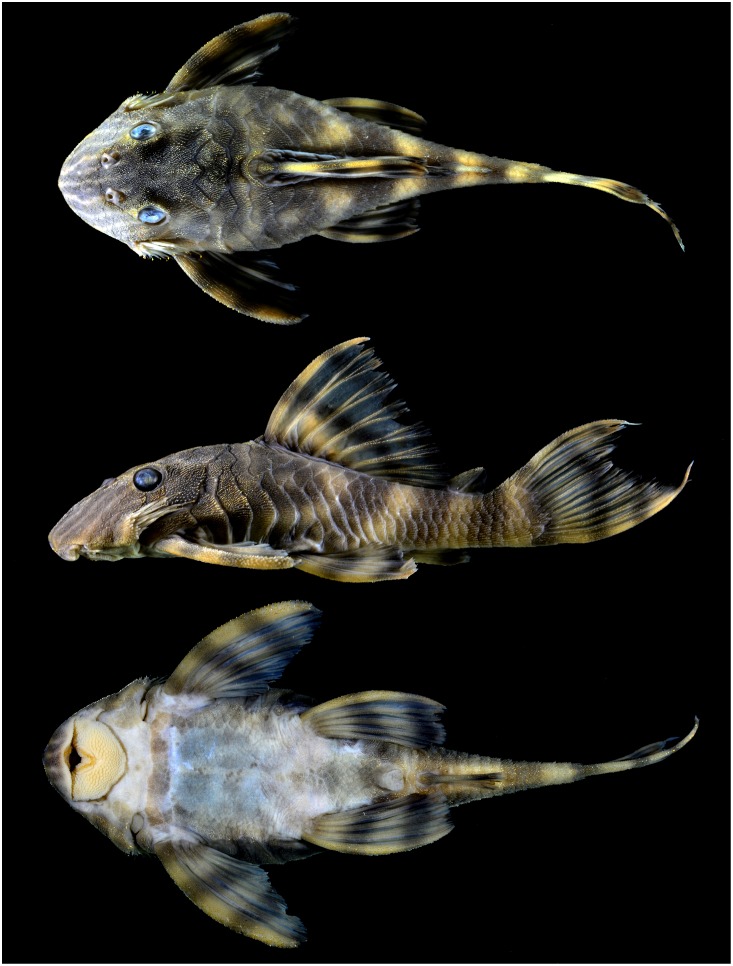
Holotype of *Panaqolus tankei* n. sp. INPA 41135, 72.8 mm SL, Brazil, Pará, rio Xingu, 17 km south from Senador José Porfírio (02°44’54.7”S 52°00’09.5”W).

*Panaqolus tankei*, new species. urn:lsid:zoobank.org:act:EA10AD3E-BD02-402F-9317-D297048B974B (Fig 1)

**Fig 2:**
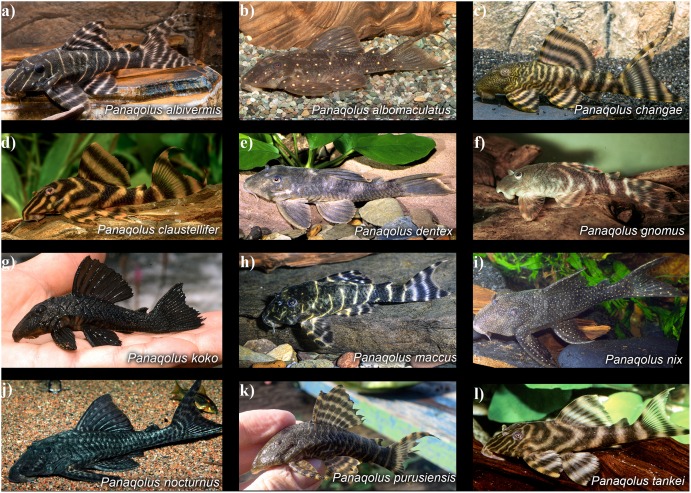
Live photos of all described species of *Panaqolus*. (photo credits: A. Tanke [a, b, c, l], R. Heidemann [d], I. Seidel [e, h], J. Dignall [f], J. Gottwald [g], H.-G. Evers [i], E. Bertelsen [j], and C. Cramer [k]).

**Fig 3:**
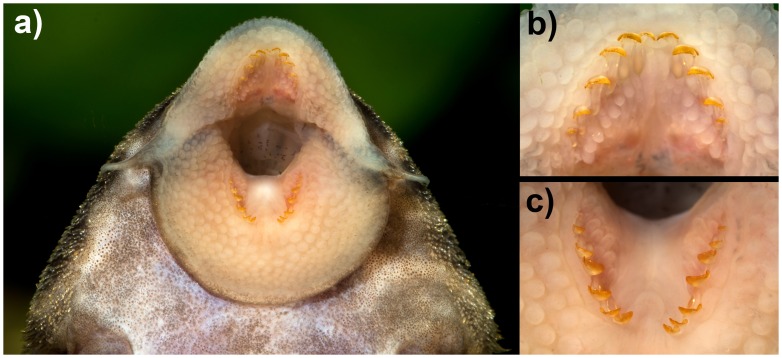
Ventral view of mouth of *Panaqolus tankei*. (a) oral disk, (b) details of premaxillary teeth and (c) dentary teeth (photos by R. Heidemann; live aquarium specimen; ca. 85 mm SL).

*Panaqolus* sp. L398: Werner [16]: 56 (Figs 1–3)

### Holotype

INPA 41135, 72.8 mm SL, Brazil, Pará, rio Xingu, 17 km south from Senador José Porfírio, main channel, (02°44’54.7”S 52°00’09.5”W), 13 Jul. 2012, L. M. Sousa et al.

### Paratypes

101 specimens. All Brazil: Pará: USNM 376497, 2, 41.6–50.2 mm SL, 72.5 km below Senador Jose Porfirio, 31.5 km above Porto de Moz (02°01’59.16”S 52°14’53.88”W), 09 Nov. 1994, L. H. Rapp Py-Daniel et al. INPA 31777, 29 (1 c&s), 26.7–82.8 mm SL, Vitória do Xingu (02°51'36"S 51°59'20"W), 04 Nov. 2008, Rapp Py-Daniel et al. MZUSP 115040, 5, 36.8–63.3 mm SL, same data as INPA 31777. MNRJ 41741, 7, 33.2–56.5 mm SL, same data as holotype. ANSP 195074, 4, 33.1–78.9 mm SL, rio Xingu, municipality of José Porfírio (02°44’54,7” S 52°0’9,5”W), 13 Jul. 2012, Sousa et al. MPEG 28832, 3, 40.6–66.9 mm SL, same data as ANSP 195074. INPA 40506, 8, 25.7–69.1 mm SL, rio Xingu, deep channel along right bank of river ca. 38 km southeast of Vitória do Xingu (3°5’32.39”S 51°44’14.1”W), 21 Sep. 2013, Sabaj-Pérez et al. INPA 40536, 8, 45.5–78.4 mm SL, rio Xingu, along left bank of river ca. 8 km east of Vitória do Xingu (2°53’18.7”S 51°56’24.54”W), 22 Sep. 2013, Sabaj-Pérez et al. INPA 40562, 17, 23.3–40.0 mm SL, rio Xingu, main channel off downstream point of large island, ca. 13.5 km east-southeast of Vitória do Xingu (2°55’16.39”S 51°53’41.6”W), 22 Sep. 2013, Sabaj-Pérez et al. INPA 40567, 6, 32.2–74.9 mm SL, rio Xingu, main channel off upstream point of large island, ca. 2.5 km northeast of mouth of igarapé leading to Vitória do Xingu. (2°51’34.2”S 51°58’46.14”W), 22 Sep. 2013, Sabaj-Pérez et al. INPA 40644, 12, 29.5–83.0 mm SL, rio Xingu, main channel along left bank, ca. 10 km southwest and across river from Senador José Porfírio (2°38’51.5”S 52°1’39”W), 24 Sep. 2013, Sabaj-Pérez et al.

### Diagnosis

*Panaqolus tankei* can be differentiated from all congeners except *P*. *albivermis*, *P*. *changae*, *P*. *claustellifer*, *P*. *gnomus*, *P*. *maccus* and *P*. *purusiensis* by the presence of dark bars on the trunk and bands on the fins alternating with light interspacing (vs. body and fins without bars or bands). It differs from *P*. *albivermis*, *P*. *maccus*, and *P*. *purusiensis* by the width of the dark bars, which is more or less the same width of the light bars (vs. dark bars at least two or three times wider than light bars) and from *P*. *changae* by the absence of vermiculation on the head (vs. vermiculation present on head). The new species differs from *P*. *claustellifer* by by the number of dorsal-fin bands (2–4 vs. 2) and their orientation being not parallel to the margin of the fin (vs. parallel to the margin), and from *P*. *gnomus* by the orientation of the bars on the body from posterodorsal to anteroventral direction (vs. anterodorsal to posteroventral direction). *Panaqolus tankei* can be further differentiated from *P*. *albivermis* by a greater cleithral width (32.2–37.0% SL vs. 29.7–32.4%), a longer head-pectoral length (25.7–32.5% SL vs. 23.1–25.2% SL), and a lower caudal peduncle depth (10.0–12.3% SL vs. 12.2–14.9% SL); from *P*. *changae* by the snout having more or less straight lines (vs. vermiculated lines in the snout) and five to seven dark bars on the trunk (vs. 6–12), the first three bars in phase of splitting, forming a D shape, or are already completely split in adults (vs. relatively thin and compact lines); and from *P*. *maccus* by the presence of alternating dark and light stripes on the head coloration (vs. light stripes frequently broken into dots, often forming a vermiculated pattern and not leading straight to the margin of the head). Live photos of all described species of *Panaqolus* are in Fig 2.

### Description

Proportional measurements in Table 1. Medium to small-sized loricariid with standard length of measured specimens up to 83.0 mm SL. Dorsal profile of head and snout strongly convex from snout tip to posterior tip of supraoccipital, straight and posteroventrally slanted between dorsal-fin origin and adipose-fin origin, gently concave through caudal peduncle to posterior tip of last procurrent caudal fin ray. Dorsal orbit margin slightly raised, forming a small ridge, narrowing anteriorly, from anterior orbit margin to area lateral to nares. Dorsal surface of trunk transversely flattened from dorsal-fin origin to adipose-fin base. Ventral profile of head and body flat from oral disk to anal-fin origin. Caudal peduncle oval in cross-section and moderately deep (10–12% SL).

**Table 1:** Selected morphometric features of *Panaqolus tankei*. Values are given as percent of standard length or head length. SD = standard deviation, n = number of specimens, H = holotype. Interlandmarks (ILM) are the two points between which measurements were taken (from [8]).

				*Panaqolus tankei*	
ILM	Measurement	H	n	range	mean±SD
1–20	Standard length	72.8	101	23.3–83.0	51.3
	**Percent of Standard Length**				
1–10	Predorsal length	48.5	83	42.5–51.1	46.3±1.6
1–7	Head length	39.2	84	31.6–45.4	38.9±1.9
8–9	Cleithral width	34.3	84	32.2–37.0	34.1±0.9
8'–9'	Cleithral width ventral	33.4	83	30.3–36.3	33.0±1.0
1–12	Head–pectoral length	26.3	83	25.7–32.5	28.4±1.4
12–13	Thorax length	27.3	84	23.0–32.1	27.0±1.6
12–29	Pectoral-spine length	34.0	84	26.3–36.5	33.0±1.6
13–14	Abdominal length	26.0	84	19.7–26.0	23.2±1.1
13–30	Pelvic-spine length	27.9	84	25.9–32.9	29.1±1.4
13–13'	Pelvic girdle width	23.8	84	19.1–24.2	22.0±0.8
14–15	Postanal length	31.2	84	23.6–34.6	31.1±1.7
14–31	Anal-fin spine length	16.9	81	10.3–19.2	14.9±1.5
10–12	Dorsal–pectoral depth	32.6	83	28.7–33.4	30.8±1.0
10–11	Dorsal-spine length	32.3	68	26.7–35.2	32.1±1.7
10–13	Dorsal–pelvic depth	26.6	82	18.6–28.0	24.8±2.0
10–16	Dorsal-fin base length	26.9	81	21.0–30.9	25.9±1.4
16–17	Dorsal–adipose distance	16.4	83	13.7–20.4	16.7±1.2
17–18	Adipose-spine length	8.5	83	5.8–11.4	8.6±0.9
17–19	Adipose–upper caudal distance	14.3	83	12.8–18.0	14.9±1.1
15–19	Caudal peduncle depth	11.2	83	10.0–12.3	11.1±0.5
20–32	Caudal peduncle–middle caudal ray	21.1	82	16.9–24.9	21.2±1.8
20–33	Caudal peduncle–dorsal caudal spine	32.0	12	32.0–44.8	38.0±4.4
15–17	Adipose–lower caudal depth	22.4	83	20.7–25.9	23.2±1.1
14–17	Adipose–anal depth	19.3	82	15.3–20.4	18.3±1.0
	**Percent of Head Length**				
5–7	Head–eye length	36.5	83	28.6–45.0	35.5±2.2
4–5	Orbit diameter	18.9	83	15.8–25.2	19.1±1.2
1–4	Snout length	59.2	83	50.9–71.6	59.2±2.8
2–3	Internares width	11.0	83	9.6–15.7	12.1±1.1
5–6	Interorbital width	51.0	83	41.4–62.9	49.7±2.9
5'–6'	Dorsal interorbital width	34.1	83	30.3–45.5	35.4±2.1
7–12	Head depth	70.3	83	58.1–80.0	68.1±3.8
1–24	Oral-disk length	44.3	83	38.1–55.9	45.1±2.5
21–22	Oral-disk width	41.1	83	35.4–49.2	41.5±2.5
22–23	Barbel length	10.1	83	7.1–16.9	11.9±2.0

Greatest body depth at dorsal-fin origin. Pectoral-fin origin just posterior to orbit, pelvic-fin origin at vertical through origin of second dorsal-fin ray, anal-fin origin shortly posterior after vertical through origin of last dorsal-fin ray. Adipose fin with well-ossified leading spine bearing odontodes.

Dorsal fin II,7, pectoral fin I,6, pelvic fin I,5, anal fin I,3–4 (mode 4), caudal fin I,14–15,I (mode 14 branched rays). Spinelet triangular, dorsal-fin spine lock functional, posterior fin margin straight, margin of last two rays rounded. Dorsal fin origin closer to vertical line passing through pelvic-fin origin than to vertical line passing through pectoral fin origin; not reaching adipose fin when adpressed. Adipose fin triangular; adipose-fin spine slanted posteroventrally, tip straight to curved ventrally, pointed; posterior margin of adipose-fin membrane concave to nearly vertical. Pectoral-fin spine robust, posterior fin margin straight, when adpressed reaching 1/3 of pelvic fin. Pelvic-fin spine robust, posterior margin slightly rounded, when adpressed reaching mid-length of anal-fin spine. Caudal fin forked with short filamentous extensions on rays.

Head and body entirely plated except for small naked area along dorsal-fin base, snout without naked area near tip. Abdomen of adults mainly naked, only triangle formed by the anus and the two pelvic-fin origins covered by small plates. Abdomen of juveniles not plated. Area dorsal to pelvic-fin base below ventral margin of inframedian plate row usually naked. Supraoccipital bordered posteriorly by 2 scutes on each side. Body with pronounced lateral ridge extending from cleithrum to posterior margin of fifth or sixth plate of the inframedian plate row; ridge gradually decreasing in prominence posteriorly. Trunk without elevated ridges. 6–7 (mode 7) scutes on dorsal-fin base, 5–6 (mode 6) scutes between dorsal and adipose fin, 5–7 (mode 6) scutes between adipose and caudal fin, 2 scutes on anal-fin base, 9–12 (mode 10) scutes between anal and caudal fin, 22–25 (mode 24) scutes on lateral line. 28 vertebrae including 5 in the Weberian apparatus and 1 the ural centra.

Head and body covered by odontodes of uniform size and distribution. Enlarged odontodes on anterodorsal border of pectoral-fin spine. Cheek odontodes hypertrophied with tips recurved distally, longest odontode extending to posterior cleithrum margin. Interorbital space straight or slightly convex. Eye moderately large, dorsolaterally placed; orbit diameter 15.8–25.2% HL. Iris diverticulum present. Nares small and ovoid, slightly longer than wide.

Oral disk more or less circular, distal margin of upper lip well separated from maxillary barbel base (Fig 3a). Maxillary barbels of moderate length (7–17% HL), tip of barbel sometimes bi- or trifurcated. Lips moderately rugose, small patch of elongate fleshy papillae medial to each tooth row. Border of lips smooth.

Teeth spoon-shaped with small lateral cusp. Premaxillary teeth 4–7 per ramus (mode 6), mandibular teeth 4–9 per ramus (mode 7). In contrast to *Panaque* [8], no correlation of SL and number of teeth was found. Premaxillae tooth rows angled at approximately 80°, dentary tooth rows at approximately 60° (Fig 3).

### Color in alcohol

**Fig 4:**
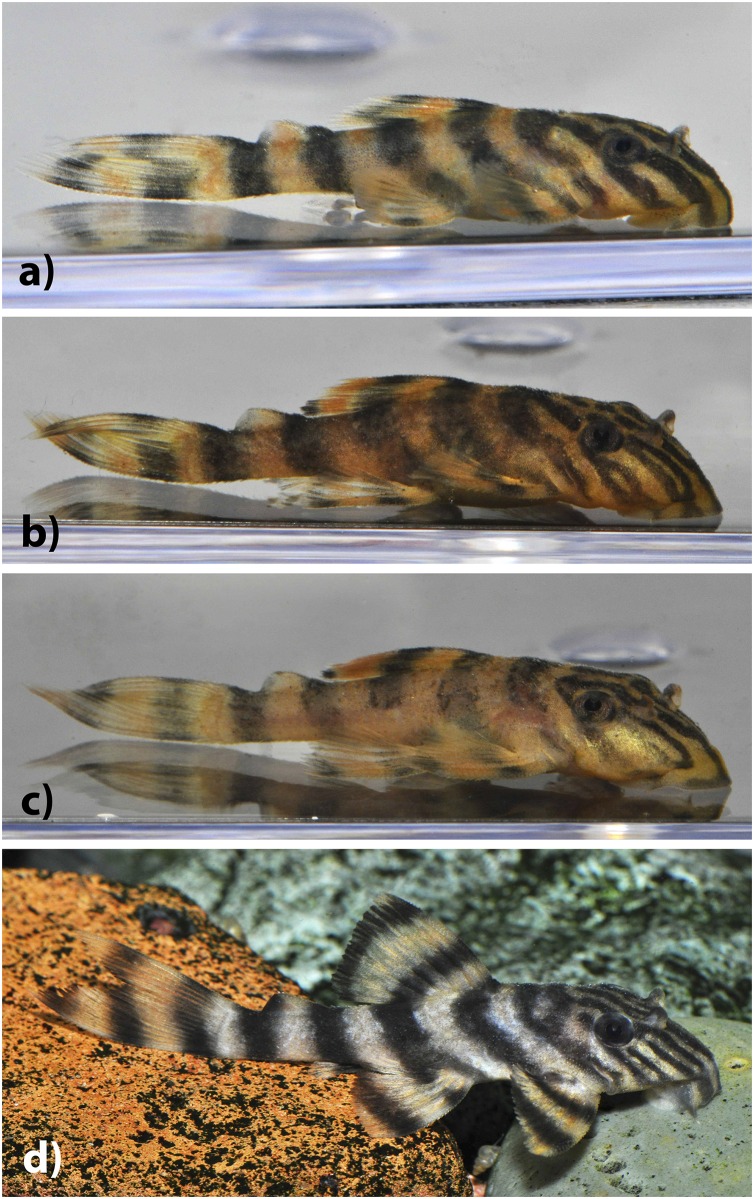
Juvenile *Panaqolus tankei*. Estimated standard lengths: (a) 14.5 mm, (b) 19.8 mm, (c) 21.4 mm, and (d) 30.0 mm (photos by I. Seidel).

**Fig 5:**
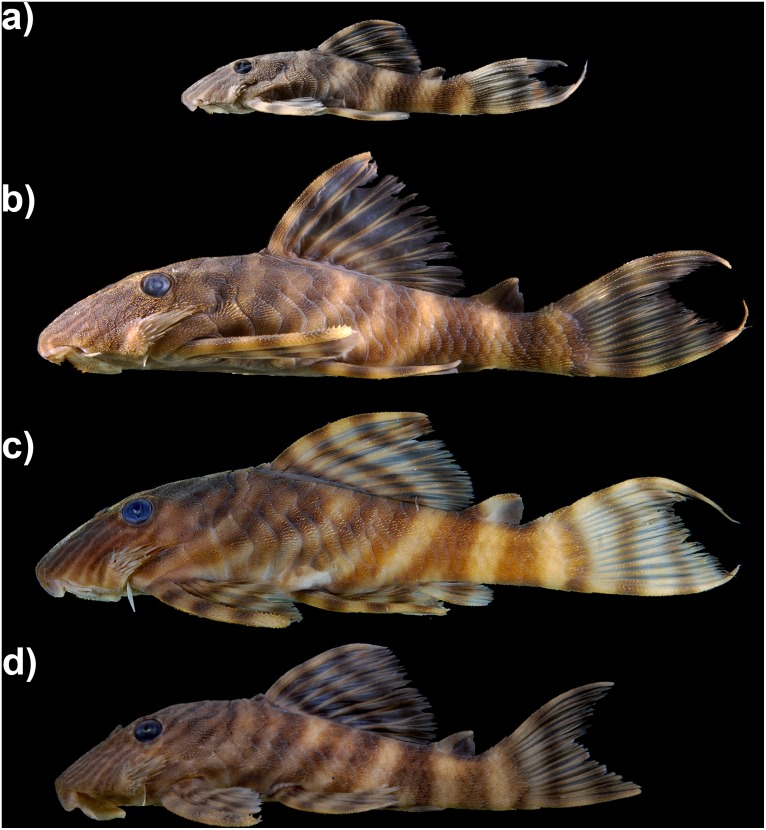
Variation in the body coloration of *Panaqolus tankei*. a) small specimen with five compact bands (ANSP 195074, 43.5 mm SL); b) adult specimen with the 1^st^ and 2^nd^ bands in phase of splitting, forming a D; the 3^rd^ band is already split into two thin bands (ANSP 195074, 78.9 mm SL); c) adult specimen with the 2^nd^ band nearly and 3^rd^ band completely split, the 4^th^ band forming an inverted Y (ANSP 195074, 73.2 mm SL); d) adult specimen with the 2^nd^ and 3^rd^ bands completely split (INPA 40644, 65.0 mm SL).

**Fig 6:**
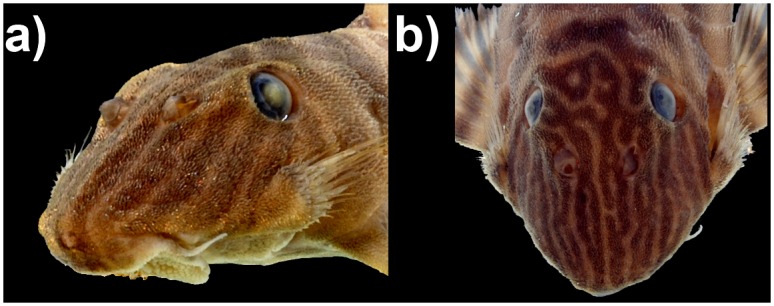
Variation of the head coloration of *Panaqolus tankei*. a) compact straight lines b) partially split lines, forming an inverted Y, an elongated 0 or a sequence of 0s (both INPA 31777).

**Fig 7:**
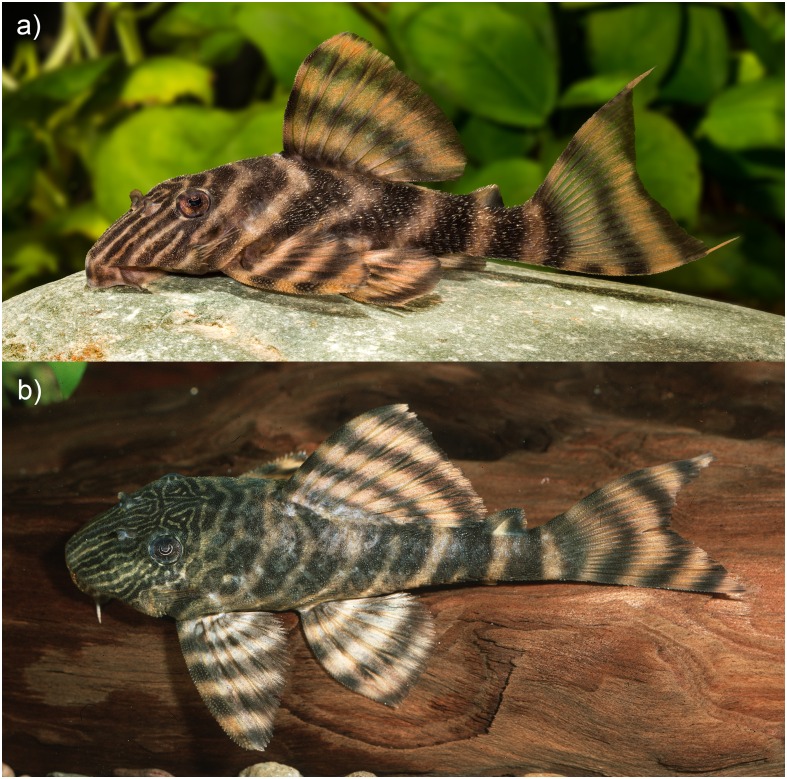
Live coloration of *Panaqolus tankei*. (a) from the region of Vitória do Xingu (live aquarium specimen; photo by R. Heidemann) and (b) from the region of Porto de Moz with more and finer lines on the head (live aquarium specimen; photo by A. Tanke).

Ground coloration yellowish white. Fins showing dark bands on light ground in all body sizes: 2–4 in dorsal fin, 2–5 in pectoral fin, 2–4 in ventral fin, 1–2 in anal fin, 2–5 in caudal fin and 1–2 in adipose fin (mode 2). Number of bands on fins increasing with body size. Small individuals have 5 compact bands (Figs 4 and 5a). In larger individuals, the first three dark bands on the body in phase of splitting, forming a D (Fig 5b), the second and third band sometimes completely split, resulting in two separate thin bands. Fourth and fifth bands sometimes in form of an inverted Y shape (Fig 5c and 5d). Head with dark lines going from supraoccipital to anterior margin of snout. 3–4 (mode 3) lines under the eye, often being split at the ventral margin of the head, forming an inverted Y shape. One dark line between eye and nare and 2 between the nares. These lines may be partially split, forming an inverted Y, an elongated 0 or a sequence of 0s (see Fig 6). Dark lines on head usually thicker than light interspaces. Belly uniformly yellowish white or with light brown dots or vermiculations. Oral disk yellowish white. Live coloration is shown in Figs 4 and 7.

### Sexual dimorphism

Mature males strongly display elongated odontodes on the caudal peduncle, whereas females and juveniles only have short odontodes.

### Distribution and habitat

**Fig 8:**
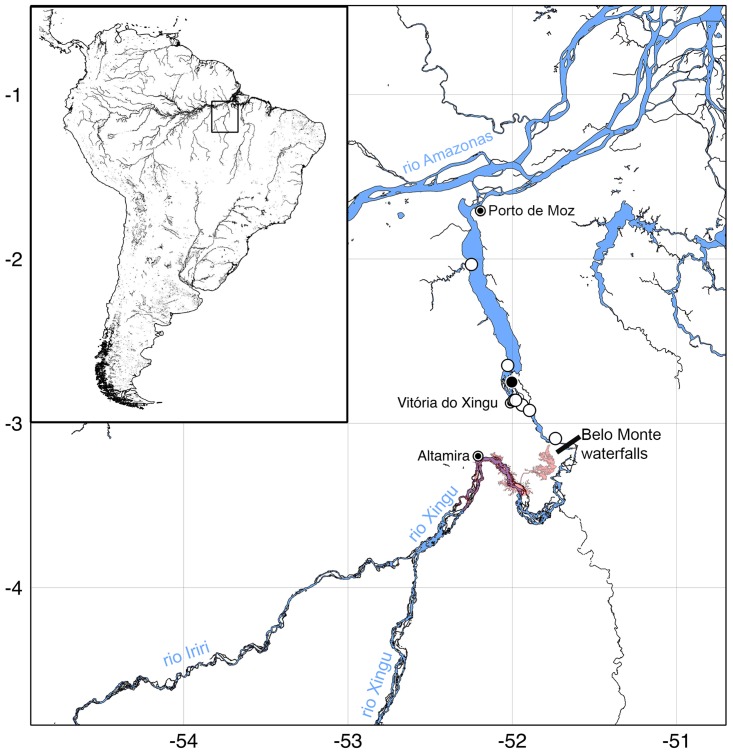
Distribution map of *Panaqolus tankei*. Solid symbol: type locality. Symbols may represent more than one locality or lot. Inset rectangle indicates location of study area in South America.

**Fig 9:**
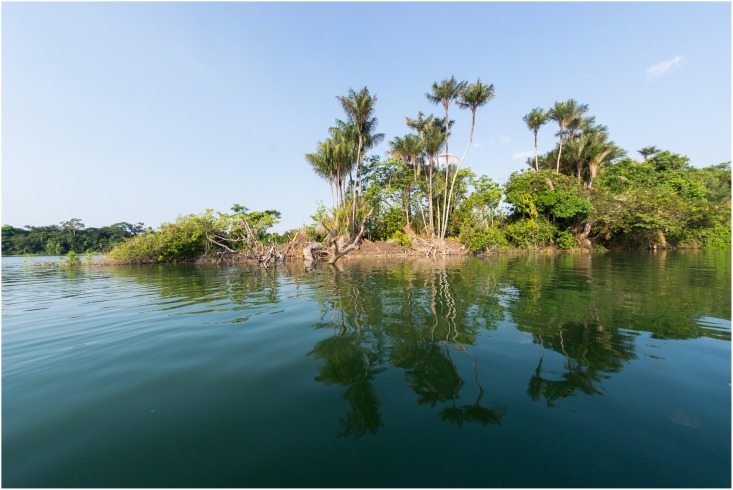
Typical habitat of *Panaqolus tankei*. Riverbank in Lower Xingu.

**Fig 10:**
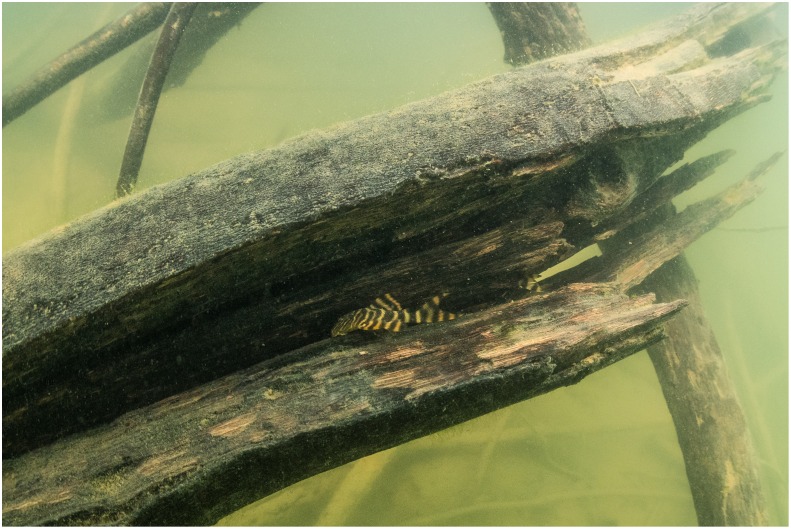
Underwater picture of the natural habitat of *Panaqolus tankei*.

The species is only known from the lower Xingu River, downstream Belo Monte Waterfalls (Fig 8). *Panaqolus tankei* are usually found on fallen trees and sunken wood alongside the riverbank, in depths varying from 1 to 10 m (Figs 9 and 10).

### Etymology

A patronym in honor of Andreas Tanke, a German aquarist very dedicated to the genus *Panaqolus*, studying its behavior, reproduction, and differences between known forms, keeping these fishes in the aquarium, visiting their habitats, and publishing his findings (e.g.[17]). He probably was the first to reproduce *Panaqolus tankei* in captivity [18]. For his (successful) efforts to improve communications between aquarists and scientists to join their forces in an era of less and less money for research and an ever accelerating destruction of natural habitats.

## Molecular Analyses

Six hundred sixty-seven (667) basepairs of the Cytochrome c Oxidase subunit 1 (COI), the so called barcode region [12], have been amplified for 12 specimens (localities, collection numbers and GenBank accession numbers in Table 2). Seven of them are *P*. *tankei*; the other five specimens belong to a putative new species from Rio Tocantins (from locations close to the cities Cametá and Portel). As only few specimens from that probable new species are available in scientific collections, analyses on its taxonomic status have not been possible. However, differences in the coloration compared to described species indicate that they might represent a new species. GenBank sequences from *P*. *changae* (EU359435.1) and *P*. *albivermis* (EU359436.1), the only congeners of which sequences were available, were used for comparison. The intraspecific variability was 0% to 1.1% for *P*. *tankei*, and 0.2% to 0.5% for the putative new species from the Rio Tocantins. Interspecific variability was 0.3% to 1.7% for *P*. *tankei-Panaqolus* sp. Tocantins, 0.8 to 1.8% for *P*. *tankei-P*. *changae*, and 4.1% to 4.6% for *P*. *tankei-P*. *albivermis*. Further distances were 0.8% to 1.1% for *Panaqolus* sp. Tocantins -*P*. *changae*, 4.1% to 4.5% for *Panaqolus* sp. Tocantins -*P*. *albivermis*, and 4.0% for *P*. *changae*-*P*. *albivermis*. The intraspecific variation in *P*. *tankei* was higher than the distances between *P*. *tankei* and the putative new species. Similar results were found in the loricariid genus *Rineloricaria*[19]. Identification via DNA barcodes is based on the observation that intraspecific genetic divergence is usually lower than interspecific divergence, leading to the so called ‘barcoding gap’[20]. At first, DNA barcoding studies suggested that a 2% threshold typically represented a cut-off between species [12], but later it was showed that the divergence between species can fall well below this value, depending on the taxa of interest [21,22]. To be able to characterize species with very low interspecific variability, a character bases approach was successfully tested [23,24]. This approach is comparable to the traditional morphology-based methods where a species diagnosis would be based on a binary signal—the presence or absence of a diagnostic character (in this case a DNA character), therefore bypassing the uncertainty found in an analogue measurement of sequence similarity. These diagnostic sites of interest are referred to as nucleotide diagnostics (ND) [24]. Such a ND was found for *P*. *tankei* in position 351 (T *vs*. A in all other analyzed species). Another ND was found for *Panaqolus* sp. Tocantins on position 198 (C *vs*. T in all other analyzed species).

**Table 2:** Locality, collection numbers, and GenBank accession numbers of the voucher specimens and the COI sequences.

Species	Tissue tag	Collection number	Locality	GenBank accession number
*Panaqolus tankei*	1132	INPA 40567	Rio Xingu, ca. 2.5 km northeast of mouth of igarapé leading to Vitória do Xingu	KT321857
*Panaqolus tankei*	1038	INPA 40506	Rio Xingu, ca. 38 km southeast of Vitória do Xingu	KT321858
*Panaqolus tankei*	1149	INPA 40562	Rio Xingu, ca. 13.5 km east-southeast of Vitória do Xingu	KT321859
*Panaqolus tankei*	1150	INPA 40562	Rio Xingu, ca. 13.5 km east-southeast of Vitória do Xingu	KT321860
*Panaqolus tankei*	1262	INPA 40644	Rio Xingu, ca. 38 km southeast of Vitória do Xingu	KT321861
*Panaqolus tankei*	1263	INPA40644	Rio Xingu, ca. 38 km southeast of Vitória do Xingu	KT321862
*Panaqolus tankei*	598	uncatalogued	Rio Xingu, Ca. 15 km downstream from Vitória do Xingu, right margin	KT321863
*Panaqolus* sp. Tocantins	P2796	MPEG31592	Rio Tocantins, 11 km upstream from Cametá	KT321864
*Panaqolus* sp. Tocantins	P2797	MPEG31592	Rio Tocantins, 11 km upstream from Cametá	KT321865
*Panaqolus* sp. Tocantins	P2807	MPEG31592	Rio Tocantins, 11 km upstream from Cametá	KT321866
*Panaqolus* sp. Tocantins	P3075	MPEG31594	Rio Camarapi, 11 km upstream from Portel, rio Tocantins drainage	KT321867
*Panaqolus* sp. Tocantins	P3079	MPEG31594	Rio Camarapi, 11 km upstream from Portel, rio Tocantins drainage	KT321868

An exhaustive comparison of the barcode region of all described species of *Panaqolus* was not possible due to difficulties in getting tissue samples. Many species occur in remote regions and are rarely caught by scientists.

## Discussion

Together with *P*. *changae*, *P*. *claustellifer* and *P*. *purusiensis*, *P*. *tankei* is part of a group of very similar species that differ mainly in details of their coloration (e.g. wormlines on the head vs. straight lines; number and width of the dark bars on the body). The forms most akin to *P*. *tankei* are a putative new species distributed in the Rio Negro basin [25] and *P*. *claustellifer*. Unfortunately, no fixed material of the former is available for comparison. Preliminary results from an analysis of DNA sequences from five genes show these (putative) species to be clearly distinct (Lujan (Royal Ontario Museum), personal communication). Furthermore, adult *P*. *tankei* have 3 to 4 dark bands in the dorsal fin which are orientated non-parallel to the margin of the fin, whereas *P*. *claustellifer* has only two bands and these are parallel to the margin of the fin [26]. Barcode sequences [12] from seven *P*. *tankei* and five specimens of *Panaqolus* sp. from rio Tocantins were very similar, with very low interspecific variation. Similar color patterns have been found for a broad variety of loricariids from the Rios Xingu and Tapajós [27]. Low genetic distances between congeners have already been reported for other members of the subfamily Hypostominae [4,19,28,29]. This pattern can also be found in other diverse groups like piranhas (*Serrasalmus* & *Pygocentrus*) [30]. This supports the theory of recent radiation of the Neotropical fish fauna [28,30].

Nucleotide diagnostics suggest that all specimens from the Rio Tocantins represent a single lineage, distinct from *P*. *tankei*. One ND could be found for each *P*. *tankei* and the new species from Rio Tocantins. Also of interest is the greater distance of all broad banded species from *P*. *albivermis*, a species with thin bands and/or dots, showing that the coloration is a strong character for differentiation in this genus. Costa-Silva et al. [19] proposed that recognition as a formal new species is justified even if only morphological traits or high molecular divergence distinguish a population from others.

From the region of Porto de Moz, near mouth of the Xingu, a population of *Panaqolus* is known which has slightly more and finer lines on the snout (A. Tanke (Neustadt am Rübenberge), personal communication; Fig 7) than *P*. *tankei* from the middle reaches of the Xingu river. As no material for comparison has been available, we cannot state here if it is a variant of *P*. *tankei* or a distinct species. Other similar populations which potentially represent distinct and undescribed species have been reported from the rivers Tocantins[31], Jari, Curuá-Una, Tapajós [32], Anapu [33], and Rio do Pará [34]. A molecular analysis is in preparation to give us further insights into the relationships between the species and populations of the genus.

Many species of *Panaqolus* are common in the aquarium trade and *P*. *tankei* has been introduced to fishkeeping hobbyists as L398 [16]. Although being an endemic species from Xingu, the geographic distribution of *P*. *tankei* does not directly overlap the impacted area of the recently implemented Belo Monte Dam. The habit of living and feeding on sunken wood in lowland riverbanks will likely remain unharmed in most parts of its distribution. However, hobbyist observations in the aquarium show that spawning of these fish appears to depend on seasonal changes in the water quality and temperature [18] and these water parameters should be constantly monitored to guarantee the natural cycles downstream the impacted area.

### Comparative material

***Panaqolus albomaculatus*: Ecuador:** USNM 167909, holotype, 91.1 mm SL, río Pucuno, tributary of Suno (altitude 350–400 m). USNM 167910, paratype, 81.7 mm SL, río Cotapino, tributary of río Pucuno. USNM 167908, paratypes, 4, 48.5–78.5 mm SL, Pastaza, río Bobonaza, Pastaza drainage. **Peru:** Amazonas: AUM 45502, 5, 39.6–103.7 mm SL, río Marañón, log riffle, 1.57 km ENE of Juan Valesco (Sta. Maria de Nieva). AUM 45507, 5, 79.1–114.8 mm SL, río Marañón, log riffle, 1.57 km ENE of Juan Valesco (Sta. Maria de Nieva). LACM 36357–33, 72.3 mm SL, río Cenepa, vicinity of Huampami. LACM 42001–9, 2, 91.4–113.3 mm SL, 100 m downstream from Caterpiza. LACM 41740–18, 99.8 mm SL, río Marañón at confluence with río Nieva at Sta. Maria de Nieva. LACM 36330–5, 120.2 mm SL, Caterpiza, quebrada. LACM 42005–10, 94.6 mm SL, 1 km upstream from Caterpiza. LACM 36313–3, 2, 84.9–96.1 mm SL, río La Poza. LACM 42115–6, 54.8 mm SL, Caterpiza. UFRO-I 17825, 77.5 mm SL, aquarium specimen. ***Panaqolus changae*: Peru:** Loreto: MUSM 17107, holotype, 58.8 mm SL, río Itaya, 11km SSW center of Iquitos at bearing 39°. SIU 29928, paratype, 45.2 mm SL, río Itaya, 11km SSW center of Iquitos at bearing 39°. INHS 42419, paratypes, 2, 38.5–83.0 mm SL, río Itaya, 11km SSW center of Iquitos at bearing 39°. AUM 28908, 5, 53.0–84.5 mm SL, río Momon, ca. 8 hours by boat from Iquitos. ***Panaqolus dentex*: Peru:** Loreto: BMNH1867.6.13.37, holotype, 58.8 mm SL, rio Xeberos, Huallaga drainage. Amazonas: FMNH 96952, 73.8 mm SL, Caterpiza. LACM 39892–1, 3, 46.9–67.7 mm SL, Shaime, village on río Yutupis, from small quebrada. LACM 41993–6, 2, 66.9–68.3 mm SL, 3 km upstream from Caterpiza. LACM 41995–3, 71.1 mm SL, 3 km upstream from Caterpiza—Kusuim. LACM 36329–6, 41.4 mm SL, Caterpiza, quebrada. LACM 41946–1, 38.3 mm SL, 200 m upstream from Caterpiza. LACM 39952–1, 4, 43.6–72.9 mm SL, Small Quebrada in Galilea, tributary to río Santiago. AMNH 218002, 76.7 mm SL, río Santiago. **Ecuador:** Napo: FMNH 97595, 2, 67.6–73.5 mm SL, Quebrada Apoalla, tributary to lower río Shushufindi, Napo drainage. FMNH 97596, 2, 74.1–78.4 mm SL, lower río Bobonaza at Chicherota, about 25 km upstream from mouth of río Pastaza. FMNH 97593, 41.4 mm SL, Estero Samonayacu, about 3.5 km SW of the bridge over río Napo along road from Coca to río Tiputini. ***Panaqolus gnomus*: Ecuador:** Pastaza: FMNH 70860, holotype, 56.5 mm SL, Cusuimi, on río Cusuimi, about 150 km SE of Puyo. FMNH 97598, paratypes, 2, 56.0–56.1 mm SL, río Bobonaza at Sarayacu, Pastaza drainage. FMNH 97597, paratypes, 3, 53.0–55.7 mm SL, Cusuimi, on río Cusuimi, about 150 km SE of Puyo. Orellana: USNM 163912, paratype, 60.9 mm SL, río Suno, upper Napo drainage. **Peru:** Amazonas: FMNH 96950, paratype, 69.2 mm SL, río Cenepa, vicinity of Huampami, elevation 700 m. LACM 42005–11, paratype, 62.0 mm SL, 1 km upstream from Caterpiza. LACM 42115–7, paratypes, 2, 59.9–67.7 mm SL, Caterpiza. LACM 41992–6, paratypes, 2, 59.3–63.6 mm SL, 500 m upstream from Caterpiza. LACM 36330–4, paratypes, 2, 59.8–63.2 mm SL, Caterpiza, quebrada. AUM 45505, 5, 51.0–64.3 mm SL, río Marañón, log riffle, 1.57 km ENE of Juan Valesco (Sta. Maria de Nieva). AUM 45501, 10, 47.0–68.3 mm SL, same data as AUM45505. ***Panaqolus koko*: French Guyana:** SMF 9702, 84.7 mm SL, Saint Laurent du Maroni: Maroni River, Saut Ga-kaba to Apatou. ***Panaqolus maccus*: Venezuela:** MCBUCV-V 24010, holotype, 66.0 mm SL, Portuguesa, río Las Marinas, upstream from bridge on Route 5 east of Cuanare, tributary of río Portugesa. FMNH 97603, paratypes, 3, 29.0–49.8 mm SL, same data as holotype. FMNH 105998, 3, 31.8–52.2 mm SL, Barinas, río Anaro, ca. 10 minutes from mouth in río Suripa, río Apuré drainage. USNM 265675, 31.6 mm SL, Bolivar, río Orinoco, Cove, Tslote de Fajardo, 182 nautic miles upstream from Sea Buoy. ***Panaqolus nix*: Brazil:** Rondônia: INPA 39606, holotype, 110.1 mm SL, rio Madeira, cofferdam at construction site of Santo Antonio hydroelectric power plant (former Santo Antonio rapids). UFRO-I 6384, 5, 50.8–80.7 mm SL, rio Marmoré, near comunity São Lourenço. UFRO-I 7968, 1, 81.4 mm SL, rio Madeira, near Ilha do Búfalo. UFRO-I 9974, 1, 53.6 mm SL, rio Madeira, below Santo Antonio rapids. UFRO-I 10050, 1, 90.4 mm SL, same data as UFRO-I 6384. UFRO-I 13039, 1, 49.5 mm SL, same location as holotype. UFRO-I 13040, 1, 57.7 mm SL, same data as UFRO-I 13039. UFRO-I 19646, 1, 97.3 mm SL, same data as holotype. INPA 39605, 3, 54.6–73.7 mm SL, same data as UFRO-I 6384. MZUSP 114009, 3, 68.5–76.2 mm SL, same data as UFRO-I 6384. Uncat., 2, 95.3–96.8 mm SL, rio Karipunas near mouth. Uncat., 1, 85.0 mm SL, rio Madeira, near mouth of rio Karipunas. Uncat., 28, 32.8–112.2 mm SL, rio Madeira, cofferdam at construction site of Jirau hydroelectric power plant (former Jirau rapids). **Peru:** ROM 92440, 1, 117 mm SL, río Tambopata, Madre de Dios drainage.

***Panaqolus nocturnus*: Peru:** Amazonas: LACM 41729–51, holotype, 138.9 mm SL, río Santiago at La Poza. FMNH 96955, paratypes, 137.8 mm SL, río Santiago at La Poza, outside mouth of quebrada by airport. LACM 41729–35, paratypes, 4, 71.3–123.4 mm SL, río Santiago at La Poza. LACM 41723–5, paratypes, 4, 111.1–123.5 mm SL, río Santiago at La Poza. AUM 45558, 6, 68.4–116.6 mm SL, río Marañón, 6.3 km NE of Juan Velasco (Sta. Maria de Nieva). AUM 45500, 2, 130.5–143.9 mm SL, río Marañón, log riffle, 1.57 km ENE of Juan Velasco (Sta. Maria de Nieva). AUM 45508, 3, 69.8–101.5 mm SL, río Marañón, 12 km N Imacita. **Ecuador:** Pastaza: FMNH 97600, paratypes, 2, 66.7–96.9 mm SL, lower río Bobonaza at Chicherota, ca. 25 km upstream from mouth with río Pastaza. Napo: FMNH 97599, paratypes, 121.5 mm SL, río Aguarico near Destacamento militar Cuyabeno and confluence of río Cuyabeno—río Aguarico, Napo drainage. USNM 167907, paratypes, 2, 103.5–109.0 mm SL, río Bobonaza, Napo drainage. USNM 177209, paratype, 92.2 mm SL, río Bobonaza at Chicherota, about 25 km upstream from mouth in río Pastaza. ***Panaqolus purusiensis*: Brazil:** Acre: AMNH 12600, holotype, 106.7 mm SL, vicinity of the mouth of rio Macauã. USNM 94665, 1, 110.6 mm SL, same data as holotype. MSNM Pi43, 1, 126.4 mm SL, same data as holotype. UFRO-I 17720, 11, 15.5–83.1 mm SL, rio Macauã near mouth with rio Iaco. UFRO-I 17723, 1, 15.7 mm SL, same data as UFRO-I 17720. MCP 35621, 2, 59.7–78.1 mm SL, rio Riozinho do Andirá at BR-364 between Rio Branco and Sena Madureira. **Peru:** Ucayali: MCP 45733, 2, 107.6–114.0 mm SL, río Curanja near confluence with río Purús. MUSM 39425, 1, 130.4 mm SL, río Curanja at mouth with río Purus. ***Panaqolus* sp.: Brazil:** Pará: MCP 44235, 2, 45.5–58.4 mm SL, rio Jari near Monte Dourado. ***Panaque armbrusteri*: Brazil:** Pará: INPA 37460, 6, 70.9–74.7 mm SL, Xingu drainage. ZMA 120.179, 1, 345 mm SL, rio Itacaiunas. ***Panaque* sp.: Brazil:** Goiás: MNRJ 13299, 7, 103.1–122.0 mm SL, rio Tocantins near Minaçu. MNRJ 13297, 1, 209.0 mm SL, rio Tocantins near Minaçu.
